# Mitigation of Salt Stress in *Lactuca sativa* L. var. Gentile Rossa Using Microalgae as Priming Agents

**DOI:** 10.3390/plants13233311

**Published:** 2024-11-26

**Authors:** Ornella Francioso, Michela Schiavon, Serenella Nardi, Davide Castellani, Erika Ferrari, Maria Teresa Rodriguez Estrada, Maria Cristina della Lucia, Veronica Zuffi, Andrea Ertani

**Affiliations:** 1 Department of Agricultural and Food Sciences, Alma Mater Studiorum, University of Bologna, Viale G. Fanin 40, 40127 Bologna, Italy; ornella.francioso@unibo.it (O.F.); dca@algaenergy.com (D.C.); maria.rodriguez@unibo.it (M.T.R.E.);; 2 Department of Agricultural, Forest and Food Sciences (DISAFA), University of Turin, Largo Paolo Braccini 2, Grugliasco, 10095 Turin, Italy; andrea.ertani@unito.it; 3 Department of Agronomy, Animals, Food, Natural Resources and Environment (DAFNAE), University of Padova, Viale dell’Università 16, Legnaro, 35020 Padova, Italy; serenella.nardi@unipd.it (S.N.); mcri.dellalucia@gmail.com (M.C.d.L.); 4 Department of Chemical and Geological Sciences, University of Modena and Reggio Emilia, Via G. Campi 103, 41125 Modena, Italy; erika.ferrari@unimore.it

**Keywords:** microalgae, biostimulant, sustainability, renewable biomass, salt stress

## Abstract

Using renewable biomass in agriculture, particularly microalgae as a biostimulant, offers economic and environmental sustainability benefits by reducing costs, improving nutrient cycling, and enhancing water use efficiency. Microalgae contain bioactive compounds that boost crop tolerance to environmental stresses, including salinity. Saline soils, characterized by elevated sodium chloride (NaCl) levels, negatively impact many crops, resulting in low productivity and high remediation costs. Therefore, this study evaluates the biostimulant properties of a microalgae-based commercial preparation (MR) on lettuce (*Lactuca sativa* L.) plants grown hydroponically and exposed to saline stress. The extract was chemically characterized through elemental analysis, lipid composition (gas chromatography with flame ionization detector—GC-FID), the determination of functional groups (Fourier Transformed Infrared—FT-IR), structure (^1^H,^13^C Nuclear Magnetic Resonance—NMR), with their hormone-like activity also assessed. Lettuce plants were treated with or without the microalgae blend, in combination with 0, 50 mM, or 100 mM NaCl. The contents of nutrients, soluble proteins, chlorophylls, and phenols, as well as the lipid peroxidation, antioxidants and root traits of lettuce plants, were estimated. The microalgae applied to salt-stressed plants resulted in a significant increase in biomass, protein, and chlorophyll contents. Additionally, significant effects on the secondary metabolism and mitigation of salinity stress were observed in terms of increased phenol content and the activity of antioxidant enzymes, as well as decreased lipid peroxidation. The potassium (K^+^) content was increased significantly in plants treated with 100 mM NaCl after addition of microalgae, while the content of sodium (Na^+^) was concurrently reduced. In conclusion, our results demonstrate that using microalgae can be a potent approach for improving the cultivation of *Lactuca sativa* L. under saline stress conditions.

## 1. Introduction

In recent years, the use of microalgae as a valuable industrial feedstock has attained increasing interest within the agricultural sector [1,2,3,4]. The appeal of microalgae lies in their ability to prosper in non-potable waters, including industrial effluents [5]. Furthermore, their capacity to efficiently modulate metabolite biosynthesis in response to various environmental conditions, along with their significant production of antioxidant molecules, enhances their attractiveness [6,7,8,9]. Indeed, various microalgae species, including *Spirulina* spp., *Dunaliella salina*, and *Chlorella* spp., have been recently reviewed [10] and acknowledged as renewable sources for food, agrochemicals, and nutraceuticals [11].

The metabolites derived from microalgae exhibit promising potential in augmenting soil fertility, bolstering plant resistance to abiotic stress, eliciting defense responses, and optimizing nutrient uptake [1,12]. However, the precise mechanisms of their action and their impact on plant physiology still lack comprehensive understanding. It is known that the growth-promoting properties associated with microalgae arise from their content in diverse biomolecules, such as amino acids, phenolics, phytohormones, terpenoids, and polysaccharides [13]. For instance, applying extracts obtained from *Chlorella vulgaris* and *Scenedesmus quadricauda* during seed hydration improved the germination rate and increased root length, root diameter, and the number of root tips in sugar beet [14]. Similar positive effects were observed when extracts of cyanobacteria species were applied to seeds of maize and wheat [15]. Moreover, the inoculation of live cyanobacteria suspensions in pepper, coriander, fennel, and cumin seeds promoted germination and enhanced the development of roots and leaves [16].

The utilization of microalgae as biostimulant to mitigate abiotic stress has become popular due to their richness in signaling and bioactive molecules, growth enhancers, antioxidants, and natural mineral chelators that support plant growth [17]. The application of extracts from *D. salina* has been shown to enhance salinity tolerance in pepper and tomato plants by increasing the activity of antioxidant enzymes [18,19]. Similarly, applying polysaccharides derived from *Spirulina platensis*, *D. salina*, *Porphyridium* spp., and *Phaeodactylum tricornutum* to tomato plants has been demonstrated to enhance phenylalanine lyase and chitinase enzyme activity, total polyphenol content, and reactive oxygen species (ROS) scavenging activity [18].

Among environmental stresses that could be mitigated by applying biostimulants to crops, salinity poses a major concern, as the amount of salinized land is progressively increasing on a global scale [20]. Areas characterized by high concentrations of sodium chloride (NaCl) salt in saline (sodic) soils pose a considerable threat to crop productivity by causing osmotic stress and toxicity in plants [21,22]. An excess of NaCl can restrict water uptake, imbalance the uptake of nutrients—particularly potassium (K^+^) and calcium (Ca^2+^)—and induce changes in protein conformation that result in alterations to plant metabolism [23,24].

There are several methods for reclaiming saline soils for crop cultivation, including deep tillage, sub-seeding, horizon mixing [25], profile inversion, washing, and leaching. The most common method for salt removal is leaching, which involves applying large volumes of water. However, due to limited water resources, alternative strategies are needed to reduce remediation costs and prevent crop productivity loss in saline environments. Eustress, a form of moderate and beneficial stress, has been increasingly studied for its potential to enhance the quality of horticultural crops, particularly vegetables. Unlike distress, which severely impacts plant health, eustress can trigger positive physiological responses, leading to improved plant performance and quality traits [26]. Among the various types of eustress, mild salt stress has gained significant attention for its role in enhancing the nutritional and functional quality of vegetables by increasing the biosynthesis of secondary metabolites, such as phenolics, flavonoids, and antioxidants [27]. For instance, moderate salinity levels have been shown to enhance the concentration of bioactive compounds in leafy vegetables like lettuce and spinach, improving their antioxidant properties without compromising yield [26,27]. Moreover, controlled saline eustress can increase the content of essential minerals such as potassium and magnesium, contributing to a better taste and nutritional profile of the produce [28,29,30].

Lettuce, a crop highly sensitive to salt, plays a crucial role in the agricultural sector [31]. Recent studies have shown that salinity can significantly disrupt lettuce’s physiological processes, particularly those associated with electron transfer in photosynthesis [32]. This disruption often leads to ionic imbalance at the cellular level and ultimately results in reduced yields. Furthermore, increasing concentrations of NaCl applied to lettuce have been reported to induce proline accumulation while simultaneously causing declines in total phenol concentration (TPC), total flavonoid concentration (TFC), ascorbic acid levels, chlorophyll content, and antioxidant activities [33]. Its cultivation and distribution are critical for supporting the livelihoods of farmers, farmworkers, and other stakeholders across the agricultural supply chain. The lettuce trade has witnessed notable growth in recent years, which has been driven by increased demand for both fresh and processed forms, resulting in expanded cultivation and export opportunities for many nations [34]. Given this scenario, contemplating the application of biostimulants in the form of microalgae blends to lettuce emerges as a potential sustainable strategy to alleviate salt stress in this horticulture crop.

So far, various microalgae extracts have been used as biostimulants to enhance crop nutrition and productivity, but limited investigation into the specific effects they induce on plant growth and productivity under salinity stress has been conducted. For these reasons, the aim of this study was to assess the capacity of a microalgae-based product to mitigate salt stress in hydroponically cultivated *Lactuca sativa* L. plants. Previous testing involved a thorough dissection and analysis of the chemical characteristics of this product. 

## 2. Results

### 2.1. Characteristics of the Microalgae-Based Product

#### 2.1.1. Elemental Analysis and Lipid Content

The elemental analyses revealed the occurrence of several macro- and microelements in the microalgae-based product (MR) (Table 1). In particular, relatively high contents of N, P, and K were determined. Among the micronutrients, the most abundant were Ca, Mg, and Fe.

The total lipid content accounted for 11% DW (dry weight), as shown in Table 2. Free fatty acids (FFAs) and diglycerides (DAGs) represented 42.30% DW and 38.50% DW of the total lipids, respectively. Additionally, hydrocarbons and carotenoids (HYCAs) were found to be 14.6% DW in the sample; in contrast, triglycerides (TAGs) and di-acylglycerols (PSTOCs) were less than 1% DW.

#### 2.1.2. Content in Amino Acids 

The MR contained a varied amino acid composition, with a predominance of certain specific ones (Table 3). Glutamic acid and leucine were among the most abundant amino acids, followed by alanine and valine. Other amino acids present in significant quantities included lysine, isoleucine, and threonine. Serine, histidine, asparagine, and glycine were also well represented in the formulation, although in slightly lower amounts. The presence of phenylalanine, tryptophan, and methionine completed the range of essential amino acids. Additionally, the product contained aspartic acid, arginine, tyrosine, proline, cysteine, and a small amount of glutamine.

#### 2.1.3. Spectroscopic Characterization of Microalgal-Based Product (MR)

In this study, different spectroscopic techniques were employed with the objective of providing a comprehensive characterization of the microalgal based product. In particular, FTIR spectroscopy is a time-efficient technique due to the simplicity of the sample preparation process, the relatively short time required for spectral analysis, and the ability to analyze only minute quantities of the sample. Furthermore, FTIR provides significant advantages such as a higher signal-to-noise ratio, high energy throughput, and high accuracy and stability. The aforementioned properties of FTIR are effective in the characterization of complex samples, such as microalgal-based products.

**Figure 1:**
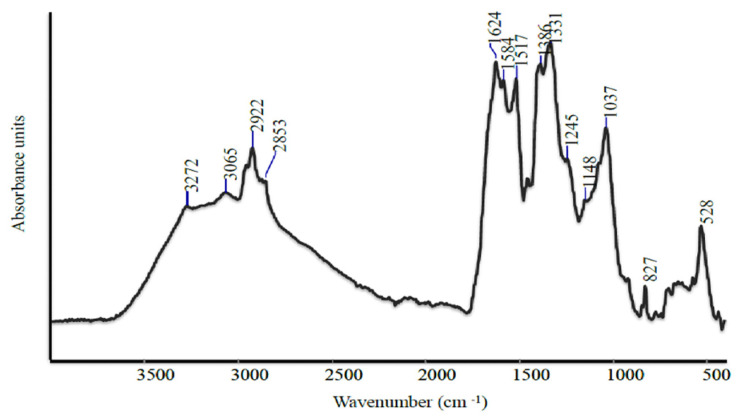
FT-IR spectrum of microalgae-based product (MR).

The FTIR spectrum of the microalgae-based product (MR) (Figure 1) showed distinct bands corresponding to different biomolecules such as lipids, carbohydrates, and aromatic compounds. In particular, the broad band at 3272 cm^−1^ was assigned to O–H stretching, while a slight shoulder at 3065 cm^−1^ was assigned to C=C–H stretching vibration and N-H stretching. The region between 2954 cm^−1^ and 2853 cm^−1^ was typical of aliphatic lipid chains [35], as well as that between 1368–1329 cm^−1^, which corresponded to the twisting vibration of -CH_2_ in C14:0–C18:0 [36]. The strong bands at 1624 cm^−1^ and the weak band at 1245 cm^−1^ were due to C=O and C–OH stretching vibration of acidic groups, respectively. The high content of FFAs might support the presence of these bands. The strong band at 1516 cm^−1^ corresponded to aromatic C=C stretching. The bands around 1037 cm^−1^ could be assigned to C–C–H and O–C–H deformation, C–O stretching, and C–O and C–C stretching vibrations of pyranose rings [37], with the contribution of mineral compounds.

**Figure 2:**
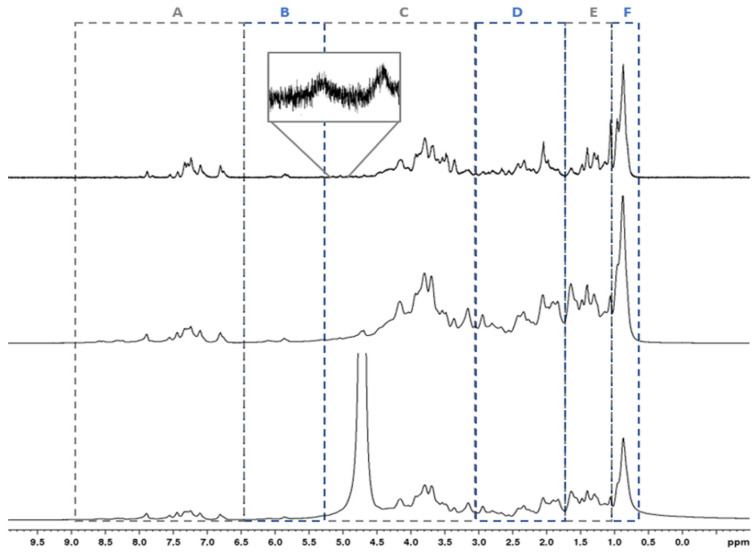
^1^H NMR spectra of microalgae-based product MR in D_2_O (298K @ 600 MHz). From bottom to top, spectra acquired using the pulse sequences: *zgcppr*, *noesypr1d*, and *cpmgpr1d*, respectively. Six typical regions are highlighted by dashed square boxes: (**A**) aromatic protons from amino acids and nucleobases (9.0–6.5 ppm); (**B**) nucleobases and double-bond (lipids and carotenoids) protons (6.5–5.3 ppm), the expansion in cpmgpr1d spectrum highlights diacylglycerol methylene group; (**C**) amino acids, carbohydrates (5.5–3.0 ppm); (**D**) methylene protons of amino acids/peptides and carboxyl-rich alicyclic molecules (3.0–1.7 ppm); (**E**) methylene protons of lipids and amino acids/peptides (1.7–1.0 ppm); (**F**) methyl protons of amino acids and lipids.

In addition to FT-IR analysis, NMR spectroscopy was used to better characterize the MR. Indeed, NMR spectroscopy is an efficient tool to investigate the chemical composition of algae and microalgae, as reported by Stirk et al. [38]. Indeed, this technique allows us to evaluate all those species that have even slight solubility in the selected solvent, D_2_O for this study, and that behave differently according to their mobility, molar mass, and wettability. As shown in the ^1^H NMR spectra (Figure 2), minimal differences were observed using 1D pulse sequences for water suppression; particularly, *cpmg* permitted the focus on “sharper” peaks due to highly mobile protons with long relaxation time, while information was lost for those protons in a fast-relaxing environment. In all cases, resonances were consistent with the presence of proteins, amino acids (AAs), carbohydrates, and lipids. The ^1^H NMR spectrum can be chunked into typical regions, as reported in Figure 2. Although overcrowded signals prevented a quantitative NMR analysis, a semi-quantitative analysis pinpointed prevalent amounts of amino acids and peptides over carbohydrates and lipids. Specifically, the integrated area corresponding to terminal aliphatic methyl groups (region F, Figure 2) was close to that for methylene groups, with an integrated area ratio approximately near 1, which suggested the presence of a little amount of long-chain lipids in comparison to amino acids. The peak around 0.9 ppm benefitted from the contribution of leucine, isoleucine, and valine—in addition to terminal lipid CH_3_. In the aromatic/double-bond regions (A + B), the presence of tyrosine (7.10–6.8 ppm), histidine (8.4–8.2 ppm), phenylalanine (7.4 ppm), and nucleobases, such as uracil (5.9/7.8 ppm), was detected and confirmed by ^1^H,^1^H COSY spectrum (Figure S1). The broad weak resonance at 5.3 ppm indicated the presence of diacylglycerol. Similarly, ^13^C NMR spectrum showed significant overlap; however, different regions could be assigned on the basis of the chemical shifts and 2D ^1^H/^13^C correlations due to scalar couplings. Definitely, the applied pulse sequence for HSQC (*hsqcedetgp*) being phase-sensitive using Echo/Antiecho-TPPI gradient selection could discriminate between CH_2_ and CH/CH_3_ cross-peaks (Figure S2).

**Figure 3:**
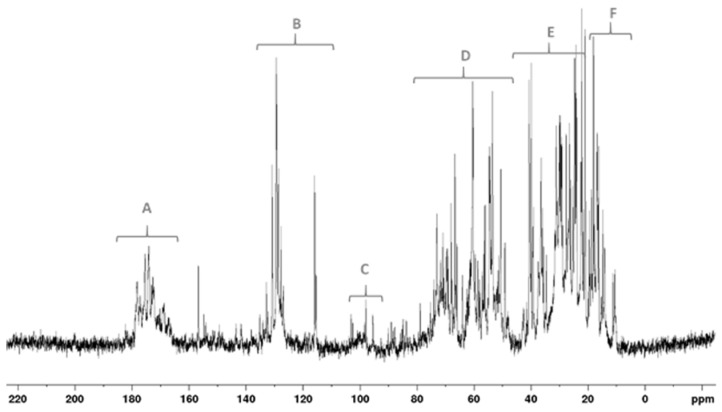
^13^C NMR spectrum of microalgae-based product MR in D_2_O (298K @ 600 MHz). Six typical regions are highlighted by dashed square boxes: (**A**) carbonyl (amides/carboxylic/esters); (**B**) aromatic carbons from amino acids and nucleobase double-bonds (lipids and carotenoids), (**C**) anomeric carbons; (**D**) amino acids/peptides/carbohydrates/diacylglycerols; (**E**) CH_2_/CH carbons of lipids and amino acids/peptides; (**F**) methyl carbons of amino acids and lipids.

As shown in Figure 3, ^13^C NMR spectrum can be divided into five main chemical shift regions associated with typically occurring compounds: FAs and protein/amino acid aliphatic side chains (0–40 ppm), protein/amino acid C alfa (40–60 ppm), C\O in carbohydrates (60–100 ppm), aromatic and double-bonded carbons (110–160 ppm), and amide and carbonyl regions (160–180 ppm). As for carbohydrate resonances, specific sub-regions were detected: 90–112 ppm for the anomeric carbon (C1), 70–80 ppm for the ring carbons (C2-5), and 60–70 ppm for the C6 carbons. This latter was also overlapped with lipids, particularly glycerol that forms mono-, di-, and triacylglycerols resonating in the same spectral region. Phase-sensitive ^1^H,^13^C HSQC analysis (Figure S2) highlighted the presence of two cross-peaks due to methylene groups tentatively attributable to diacetyl-glycerol, respectively, at δ 3.95(^1^H)/63.9(^13^C) and 3.8(^1^H)/66.2(^13^C) ppm [39]. Triacylglycerols were not observed, probably due to their poor water solubility. Carboxylic groups of free fatty acids were detected around 173 ppm. At higher chemical shifts (180–174 ppm) the contribution of amide groups was observed, while at lower chemical shifts (~170 ppm), esters were present. The signals in the range 150–160 ppm were possibly due to tyrosine and arginine residues.

The overlapping of different species hindered quantitative analysis. Nevertheless, the low intensity of anomeric carbons suggested a minimal contribution of carbohydrates, with proteins and amino acids being the main constituents. The absence of a very intense and broad peak around 20 ppm accounted for a low content of long-chain hydrocarbons and lipids, although their presence could be underestimated because of their low solubility in the aqueous medium.

### 2.2. Hormone-like Activities

**Figure 4:**
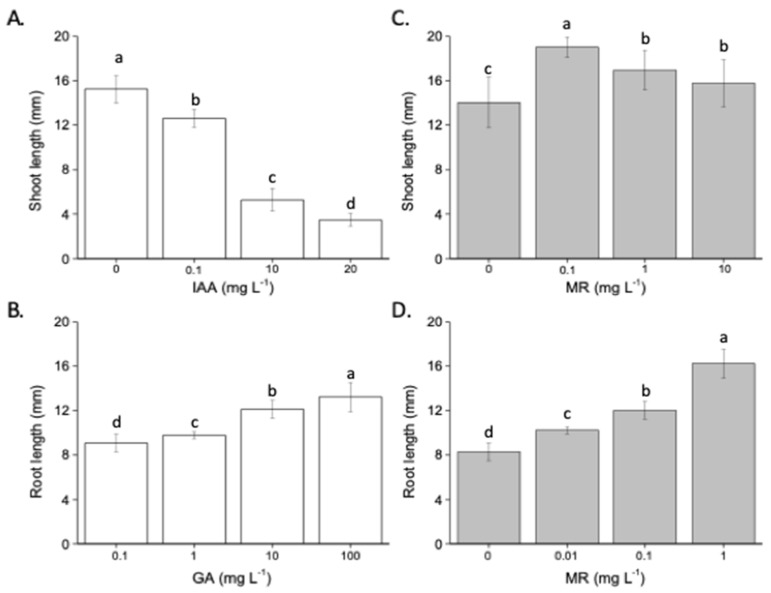
Auxin-like activity of Indoleacetic acid (IAA) (**A**) and MR (**C**), and gibberellin-like activity of gibberellic acid (**B**) and MR (**D**) supplied at increasing doses. Data represent the means of three measurements with ten plants in each. Values above bars following the same letter are not statistically different at *p* < 0.05 according to the Student–Newman–Keuls test.

The hormone-like activity of the product was assayed to determine if the presence of certain compounds endowed in the formulation could exhibit the capacity to stimulate plant growth through this hormone-regulated mechanisms. The IAA concentrations applied during the bioassay inhibited the root elongation of watercress in a dose-dependent manner (Figure 4A). In contrast, this dose-dependent inhibition did not occur when the microalgae-based product—MR—was applied at watercress seedlings. Conversely, GA concentrations promoted the elongation of lettuce shoots in a dose-dependent manner (Figure 4B), and a similar trend was observed when the microalgae-based product was applied, suggesting an effect on shoot elongation consistent with the GA-like activity of the MR.

### 2.3. Effect of MR on Root Morphology

To evaluate the effectiveness of the MR in stimulating root growth in lettuce, various root morphological parameters were analyzed and compared between MR-treated and untreated plants (Table 4). The results revealed overall stimulation of the root system. The projected area (cm^2^), which is classified as the two-dimensional area measurement of a three-dimensional object projecting its shape onto an arbitrary plane, indicated a stimulation increase of about 13% in the MR-treated plants compared to the control. A similar rate of increase was calculated for the surface area, which takes into account the entire three-dimensional surface of the root and showed a 13% increase compared to the control. The surface area of a solid object is mathematically defined as a measure of the total area that the root’s surface occupies in a given space. The length per volume, referred to as the ratio of root length (cm) to the volume of soil (m^3^), indicated a considerably higher growth rate (+47%) in plants treated with the MR. On average, the data recorded for average diameter (mm) and root volume (cm^3^) showed lower values than the control conditions. The number of root tips, forks, and crossings showed increases of 26%, 28%, and 66%, respectively, in MR-treated plants compared to untreated plants.

### 2.4. Effects of the Microalgae-Based Product and NaCl on Leaf and Root Biomass, Chlorophyll and Protein Content

**Figure 5:**
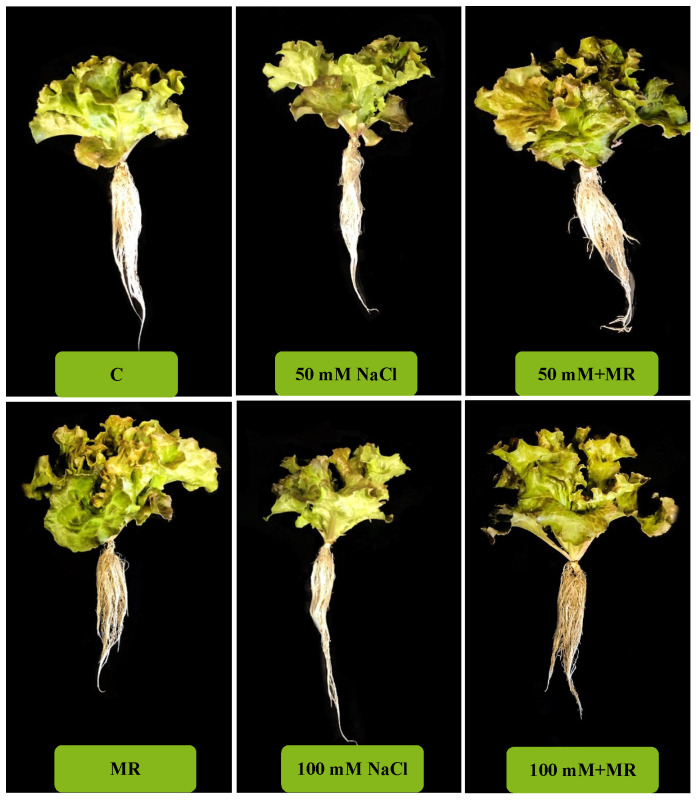
Plants of *Lactuca sativa* L. grown for 14 days in a complete culture solution with 0 (control), 50, and 100 mM NaCl and microalgae-based product (MR).

**Figure 6:**
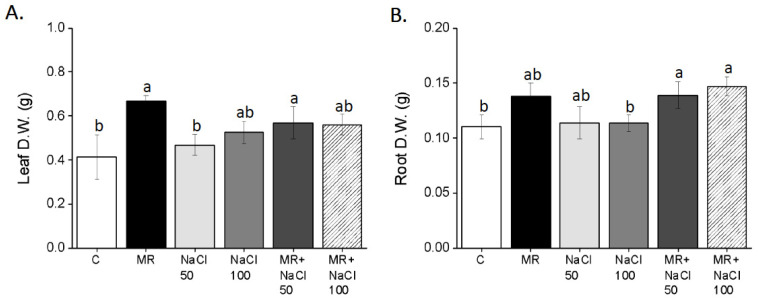
Leaves (**A**) and roots (**B**) dry weight of *Lactuca sativa* L. plants grown for 14 days in a complete culture solution with 0 (control), 50, and 100 mM NaCl and microalgae-based product (MR). All data represent the means of five measurements per treatment with three plants in each (±SD). Different letters in the same column indicate significant differences between treatments (*p* < 0.05) according to the Student–Newman–Keuls test.

Figure 5 illustrates the growth of lettuce plants at the end of the trial before harvesting. A significant increase in leaf dry biomass was observed in plants treated with MR, either alone or in combination with 50 mM or 100 mM NaCl, compared to untreated plants (Figure 6A). 

Similarly, the addition of MR to plants grown with NaCl concentration increased the leaf and root biomass (Figure 6B). Lettuce plants treated with salt at both concentrations showed no significant changes in leaf and root biomass compared to the control. 

**Figure 7:**
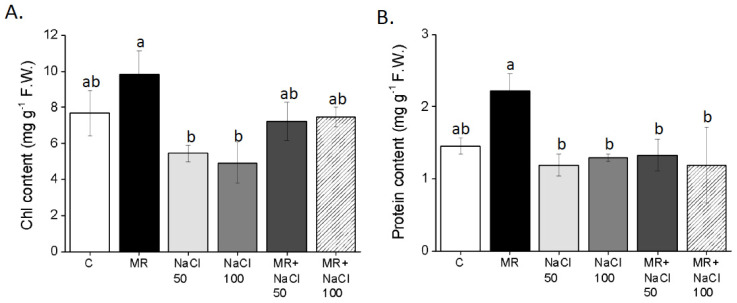
Chlorophyll (**A**) and protein (**B**) content of *Lactuca sativa* L. plants grown for 14 days in a complete culture solution with 0 (control), 50, and 100 mM NaCl and microalgae-based product (MR). All data represent the means of five measurements per treatment with three plants in each (±SD). Different letters in the same column indicate significant differences between treatments (*p* < 0.05) according to the Student–Newman–Keuls test.

The leaf chlorophyll content was substantially decreased by NaCl application compared to the control plants (Figure 7A), while the microalgae-based product (MR) enhanced it. The application of the MR to NaCl-treated plants restored chlorophyll levels, with values similar to those observed in the control group and in MR salt-untreated plants. A significant increase in total protein content was observed in plants treated with MR (Figure 7B), but MR did not improve the protein content in plants exposed to salt stress, which exhibited similar protein levels compared to the control plants.

### 2.5. Effects of the Microalgae-Based Product on Total Phenol Content 

**Figure 8:**
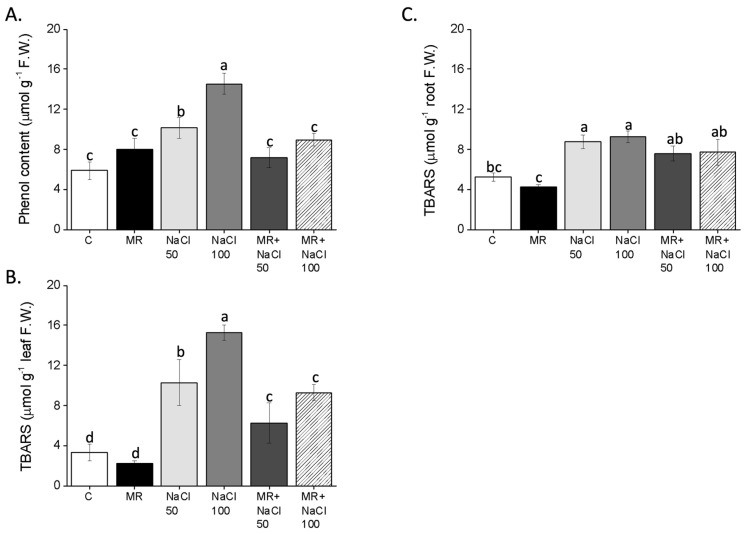
Phenols content in leaves (**A**) and lipid peroxidation in leaves (**B**) and roots (**C**) of *Lactuca sativa* L. plants grown for 14 days in a complete culture solution with 0 (control), 50, and 100 mM NaCl and microalgae-based product (MR). All data represent the means of five measurements per treatment with three plants in each (±SD). Different letters in the same column indicate significant differences between treatments (*p* < 0.05) according to the Student–Newman–Keuls test.

Overall, all treatments resulted in an increase in phenol content compared to the control, with the higher concentration of salt yielding the greatest effect. Specifically, plants treated with MR, in combination or not with 50 mM NaCl, showed a slight increase in phenol content compared to the control (Figure 8A). The addition of MR to plants treated with 100 mM NaCl decreased the amount of phenols by about 40% compared to plants treated with NaCl alone.

### 2.6. Effects of the Microalgae-Based Product on the Accumulation of Na^+^ and K^+^

The K^+^ content in the leaves was comparable between control plants and those supplemented with the MR (Table 5). The NaCl treatments led to an increase in leaf K^+^ accumulation, which was further amplified by the addition of MR. In the roots, K^+^ content was higher in NaCl-treated plants compared to the control and MR groups. Nevertheless, the MR treatment improved K^+^ retention in the roots relative to plants treated with NaCl alone. Regarding the Na^+^ content, it was significantly elevated in both leaves and roots of plants treated with NaCl (Table 5). 

The addition of MR reduced Na^+^ accumulation in both leaves and roots compared to NaCl-only treatments. The K^+^/Na^+^ ratio was highest in control plants and decreased in NaCl-treated plants. The addition of MR improved the K^+^/Na^+^ ratio in both leaves and roots under salt stress conditions.

### 2.7. Effects of the Microalgae-Based Product and NaCl on Lipid Peroxidation and Antioxidants 

The addition of NaCl, particularly at the 100 mM concentration, significantly increased total phenol production, while MR supplementation resulted in only a slight increase compared to the control plants (Figure 8A). However, the MR reduced the phenol content in NaCl-treated plants to levels similar to those in control plants. 

Lipid peroxidation, as indicated by the MDA assay, was significantly higher in the leaves of lettuce plants treated with 50 and 100 mM NaCl compared to both control plants and MR-primed plants not exposed to salt (Figure 8B). The MR substantially decreased (by about 60%) lipid peroxidation in the leaves of lettuce exposed to NaCl. In the roots, a similar trend was observed as in the leaves, although the reduction in lipid peroxidation due to MR priming was less pronounced (Figure 8C).

**Figure 9:**
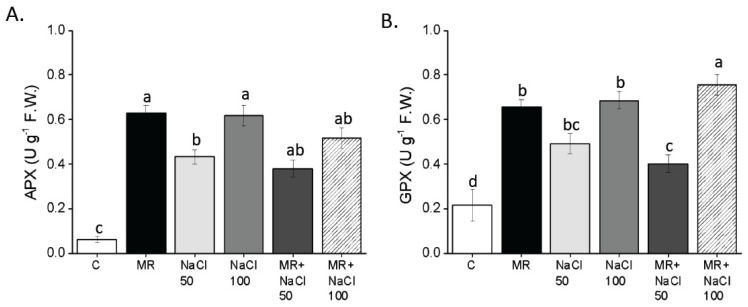
APX (**A**) and GPX (**B**) in roots of *Lactuca sativa* L. plants grown for 14 days in a complete culture solution with 0 (control), 50 and 100 mM NaCl and microalgae-based product (MR). All data represent the means of five measurements per treatment with three plants in each (±SD). Different letters in the same column indicate significant differences between treatments (*p* < 0.05) according to the Student–Newman–Keuls test.

Regarding APX enzyme activity, all treatments showed a significant increase compared to the control (Figure 9A). In particular, the treatments with the MR and 100 mM NaCl resulted in the most significant increases. The combination of the MR and NaCl did not further increase the activity of APX in plants. The trend observed for GPX was the same as for APX (Figure 9B). 

## 3. Discussion

Plants constantly face various adverse environmental stress conditions, with soil salinity posing a significant global challenge for agricultural production [40,41]. Ongoing efforts aim to develop solutions that help crops mitigate salt-induced stress, including the production of valuable products to enhance plant resilience. In this study, we specifically evaluated the effectiveness of a microalgal blend as a priming agent with biostimulant properties in lettuce plants subjected to two concentrations of NaCl. These concentrations were chosen based on previous studies [32,42]. Overall, the chemical characterization of the microalgal blend confirmed the antioxidant properties of the MR product, being in line with previous reports [43,44]. The MR demonstrated positive effects on various parameters of lettuce. These benefits were likely due to the bioactive compounds contained in the formulation [4,45,46,47], some of which are gibberellins [48] that may mimic (GA)-like activity, as suggested by the Audus test, thereby improving plant growth and root traits [8,49,50]. In addition, the lipid composition of the blend was characterized by a high presence of DAGs, FFAs, and HYCA, which are all known for their bioactive properties in plants and for their involvement in cellular defense systems against stress. These compounds may synergistically enhance plant resilience and development, promoting improved physiological responses and growth outcomes regardless of environmental stressors. DAGs, for instance, serve as secondary messengers in plant cells, being involved in signaling pathways that regulate growth and stress responses. Algal-derived DAGs may enhance plant signaling mechanisms that control responses to environmental stresses such as salinity, and they may influence the synthesis of hormones, especially abscisic acid, which is required in osmotic stress regulation [47]. FFA, including phytosterols and tocopherols, were the most abundant lipid components in the blend. These compounds are known for their capacity to improve plant cell membrane stability and regulate hormone signaling pathways under stress conditions. Tocopherols, in particular, are potent lipid-soluble antioxidants that protect plant cells from oxidative stress. The HYCA fraction, which was the third most abundant lipid component, contains carotene. This compound has well-known antioxidant properties, protecting plant cells from oxidative damage [51,52]. Regarding the content of amino acids, glutamate, leucine, and alanine were the most abundant in the MR. In recent years, some reports have noted that the exogenous application of amino acids can protect plants from abiotic stresses, including glutamate [53,54] and leucine [55], while the effects of alanine has not yet been clarified in this context [55]. On the other hand, the content of proline, widely recognized as the most significant osmolyte for mitigating osmotic stress under salinity [56], was relatively low in the product. This suggests that its contribution to the product’s beneficial properties was likely minimal.

When NaCl was applied to the plants, we did not observe significant negative effects of NaCl on plant growth and protein content compared to the control group (i.e., the non-salt-treated plants). These findings differ from those of other studies, where similar salt concentrations often resulted in reduced plant growth [31,33,57]. One possible explanation is that the lettuce variety used in the study may have been relatively salt-tolerant, as suggested by its capacity to enhance K^+^ accumulation in the leaves and roots in response to salt exposure. Additionally, this variety demonstrated a substantial increase in the activity of two key antioxidant enzymes, APX and GPX, with the effect being even more pronounced at higher NaCl concentrations, where lipid peroxidation was more evident. The activity of these enzymes typically serves to counteract NaCl-induced oxidative stress [58]. Thus, while the application of the MR to non-salt-treated lettuce plants significantly improved growth, chlorophyll and protein content, and antioxidant enzyme activity, its addition to salt-treated plants did not further significantly enhance these parameters, except for leaf growth. On the other hand, it is important to note that lipid peroxidation decreased in salt-treated plants supplemented with MR, likely due to the microalgal blend ability to considerably restrict Na^+^ accumulation in lettuce and concurrently increase K^+^ intake and the K^+^/Na^+^ ratio. The reduced accumulation of Na^+^ in these plants following MR application likely resulted in lower stress levels, which may explain the decreased production of phenolic compounds, typically acting as non-enzymatic cellular antioxidants, as well as the lack of a cumulative effect on antioxidant enzyme activity [59,60]. The reduction in stress was further supported by the lower levels of lipid peroxidation, which, under high-stress conditions, would otherwise compromise cell membrane integrity [47]. We cannot however exclude that some constituents of MRs with antioxidant properties, such as tocopherols and carotenes, may directly contribute to safeguarding lettuce cells against peroxidation and maintaining cellular redox status [48]. 

## 4. Materials and Methods

MR*, the commercial biostimulant preparation, was produced and provided by AlgaEnergy ((Madrid, Spain)) as an enzymatic digestion of microalgae extracts stabilized at specific pH values for conservation. The entire production process, from cultivation conditions to the final product, is safeguarded by patent protection. Further insights into its composition and elaboration process, via UPT^®^, can be obtained upon request to the provider.

### 4.1. Chemical Characterization of the Microalgae-Based Product

#### 4.1.1. Elemental Analysis, Quantification of Moisture and Ashes

The elemental characterization was performed on the lyophilized microalgae-based product, termed MR. Total carbon (C) and nitrogen (N) contents were determined using a dry combustion procedure inside an element analyzer (CHNS-O EA 1110 Thermo Fisher Scientific, Waltham, MA, USA). The contents of macro- and microelements were quantified via microwave-assisted acid digestion (Milestone, Shelton, CT, USA) using HNO_3_ suprapur (Carlo Erba Reagents Srl, Milan, Italy) and H_2_O_2_ (30% *v*/*v*). The mineralized samples were then diluted with ultrapure water, and each element was analyzed by Inductively Coupled Plasma–Optical Emission Spectroscopy (ICP-OES) (Spectro Amatek Arcos II ICP-OES, Kleve, Germany).

Moisture and ash content were determined using a TG-DTA92 instrument (SETARAM, Caluire-et-Cuire, France). A quantity of 4–5 mg of lyophilized sample was accurately measured and placed in an alumina crucible. The sample was hence subjected to heating, ranging from 30 to 700 °C, within a dynamic air atmosphere (air flow: 5 L h^−1^). The heating rate was 10 °C min^−1^. All analyses were conducted in triplicate.

#### 4.1.2. Analysis of Functional Groups and Structure via Attenuated Total Reflectance (ATR-IR) and Nuclear Magnetic Resonance (NMR) Spectroscopies

The Fourier Transformed Infrared (FT-IR) spectrum of lyophilized MR was recorded with an Alpha FT-IR instrument (Bruker Optics, Ettlingen, Germany). The spectral range was set between 4000 and 400 cm^−1^, with a resolution of 4 cm^−1^ and 64 scans. Prior to sample measurement, a background spectrum was recorded against air. Less than 1 mg of freeze-dried sample was used [49].

The NMR spectra were recorded using a Bruker Biospin FT-NMR AVANCE III HD (Bruker, Billericca, MA, USA) (600 MHz) spectrometer equipped with a CryoProbe BBO H&F 5 mm in inverse detection. Nominal frequencies were 150.90 MHz for ^13^C and 600.13 for ^1^H, respectively. Fifteen mg of the investigated lyophilized microalgae-based product were suspended in 0.6 mL of D_2_O (99.8%) and dropped into a 5 mm NMR tube. All experiments were performed at 298 K. A 90° (π/2) pulse was calibrated, and standard NMR parameters were used. As a result, 1D ^1^H NMR data were acquired using standard Bruker pulse sequences: *zg* (1D sequence), *zgcppr* (1D sequence with presaturation and using composite pulse for selection), *noesypr1d*, and *cpmgpr1d* (Carr-Purcell-Meiboom-Gill). These 1D ^13^C NMR spectra were acquired using Bruker pulse sequences *zgpg_pisp_f2.fas*. Typical 2D homo- and heteronuclear techniques were used for assignment, applying Bruker library pulse sequences, namely, *cosygpprqf* (^1^H,^1^H-COSY), *hsqcedetgp* (^1^H,^13^C-HSQC), and *hmbcgplpndqf* (^1^H,^13^C-HMBC) [61,62].

#### 4.1.3. Lipid Content

A modified version [49] of the Folch method [50] was used for lipids extraction from 8 g of the lyophilized microalgae-based product. The lipid content was quantified gravimetrically and expressed as percentage. Three independent replicates of the sample were analyzed. The qualitative profile of the main lipid classes (hydrocarbons + carotenes, HYCA; free fatty acids, FFA; monoacylglycerols, MAG; phytosterols + tocopherols, PSTOC; di-acylglycerols, DAG; triacylglycerols, TAG) was determined by gas chromatography–flame ionization detection (GC-FID) according to Toschi et al. [51].

An aliquot of 30 mg from the lipid extract was dissolved in 1 mL of n-hexane and injected into a GC-FID Shimadzu GC 2010 plus (Kyoto, Japan), which was coupled with a fused silica capillary column (Mega SE52 (Legnano, Milan, Italy); 10 m × 0.32 mm i.d. × 0.1 μm film thickness). The temperature program of the oven ranged from 100 to 355 °C at 5 °C min^−1^, which was held at 355 °C for 15 min. Both injector and FID temperatures were set at 355 °C. The injection was performed in the split mode (1:25), with a constant helium flow (carrier gas) of 2.02 mL min^−1^. The lipid classes were identified using different commercial standards, and the data are expressed as area percentage values. Two independent replicates of the sample were analyzed.

#### 4.1.4. Amino Acid Composition

The free amino acid content in plant samples was analyzed following the method outlined by [52]. Two grams of sample was transferred into a centrifuge tube. An internal standard (1.7-diaminoheptane solution, Merck, Darmstadt, Germany) was added to the sample to facilitate accurate quantification. Subsequently, 40 mL of trichloroacetic acid (5% *w*:*v*, Avantor Performance Materials, Gliwice, Poland) was added to the sample, which was homogenized by agitation. After centrifugation at 10,000× *g* for 10 min, 100 µL was added with 2.5 mL of di-sodium tetraborate (3% water solution, J.T. Baker, Pol-Aura, Gliwice, Poland) and 2.5 mL of dansyl-chloride (20 mM in acetonitrile, abcr GmbH, Karlsruhe, Germany) to allow derivatization of the amino acids. Derivatization was achieved by shaking the mixture in a water bath at 40 °C for 1 h. To halt the derivatization process, 10 µL of formic acid (98–100% purity, Avantor Performance Materials, Gliwice, Poland) was added to the mixture. Finally, the derivatized sample was filtered into a chromatographic vial using a syringe filter (pore size: 0.22 µm, Captiva Econofilters, Agilent, Santa Clara, CA, USA) in preparation for subsequent chromatographic analysis.

Ultra-high performance liquid chromatography coupled with a high-resolution mass spectrometer (UHPLC-MS/MS) analysis was conducted to determine the concentration of free amino acids in the samples. Chromatographic separation of the compounds was achieved using a Cortecs UPLC C18 column (2.1 × 100 mm, 1.6 µm particle size, Waters, Milford, MA, USA). The mobile phase consisted of two phases: Phase A comprised water with acetonitrile (90:10) containing 0.1% formic acid (FA) and 5 mM ammonium formate, while Phase B comprised acetonitrile with water (90:10) containing 0.1% FA and 5 mM ammonium formate. Both LC-MS-grade water and LC-MS-grade acetonitrile were sourced from Witko (Łódź, Poland), while formic acid (98–100%) and ammonium formate (≥97%) for LC-MS were obtained from Chem-Lab (Zedelgem, Belgium). A liquid phase gradient was applied at a flow rate of 0.3 mL/min, with the following composition over time: 0–2 min: 90% A, 10% B; 2–22 min: 0% A, 100% B; 22–25 min: 0% A, 100% B; 25–26 min: 90% A, 10% B; 26–28 min: 90% A, 10% B. The UHPLC-MS/MS system utilized a Q Exactive Orbitrap Focus MS (Thermo Fisher Scientific, Waltham, MA, USA) equipped with a heated electrospray ionization (HESI) source. The analysis was performed in positive polarization mode, with an injection volume of 2.5 µL. Specific MS parameters were set as follows: spray voltage: 3 kV, capillary temperature: 256 °C, sheath gas flow rate: 48, auxiliary gas flow rate: 11, sweep gas flow rate: 2, probe heater temperature: 413 °C, S-lens RF level: 50, resolution: 70,000 in simultaneous scan and 35,000 in all ion fragmentation. Data acquisition and analysis were conducted using Xcalibur 4.2.47 software, facilitating precise determination and quantification of free amino acids in the plant samples.

#### 4.1.5. Assessment of Hormone-like Activities

Indoleacetic acid (IAA)-like activity was estimated by assessing the reduction of roots of watercress (*Lepidium sativum* L.) after treatment with IAA and different aqueous solutions of the products, while gibberellin (GA)-like activity was evaluated by measuring the increase in length of lettuce epicotyls after treatment with increasing doses of GA and the microalgae-based product. The detailed protocol is reported in Ertani et al. [53].

### 4.2. Experimental Design

Two-week-old plantlets of *Lactuca sativa* L. var. gentile rossa were transplanted from the potting soil into 6 L hydroponic pots (35.5 × 12.5 × 21.5 cm) filled with a Hoagland modified nutrient solution, at a density of 6 plants per pot, which was renewed every 48 h. The nutrient solution had the following composition: KH_2_PO_4_ (0.63), Ca(NO_3_)_2_ (2), KNO_3_ (3), MgSO_4_ (1.5), FeNaEDTA (0.040), plus micronutrients. Before the transplant, the roots of plants were thoroughly washed with deionized water to gently remove the soil particles. Lettuce plants were cultivated inside a growth chamber at 22 ± 2 °C, with a relative humidity ranging within 70%–85% and a 16 h photoperiod under natural light. Seven days after transplantation, the microalgae-based product was added to the nutrient solution of half of the plants, at a concentration of 1mL L^−1^, according to literature tests. This addition was done to induce a priming effect in the plants before subjecting them to the salt stress treatment. Two days later, lettuce plants, primed or not with the microalgae-based product, were treated with the following NaCl concentrations: 0, 50, and 100 mM. Three pots were set up for the control plants (unprimed and salt untreated) and each treatment (18 pots in total). Plants were sampled 5 days after salt treatment, divided into roots and leaves, and carefully washed. Sub-samples from control and treated plants were frozen with liquid nitrogen and stored at −80 °C. The fresh weight (FW) was determined for leaves and roots of 9 plants. Roots and leaves samples were then dried at 70 °C for 2–3 days, and their dry weight (DW) was determined. The experimental design is represented in Figure S3.

### 4.3. Root Morphological Analysis

Root morphological analysis was performed by using the WinRHIZO software 2012 (Regent Instruments). Total root length, root surface area, mean root diameter, root volume, total number of root tips, lateral root (0.000 < L < 0.500 and 0.500 < L < 1.000) were determined by computerized scanning (STD 1600, Regent Instruments, Québec, QC, Canada) [54,55].

### 4.4. Elemental Analyses

Dried leaves and roots of lettuce plants were ground before elemental analysis. The digestions were carried out as described in Ertani et al. [54] inside closed Teflon vessels of 120 mL volume using approximately 500 mg dry leaf or root material and 10 mL of 30% (*v*/*v*) HCl. After digestion, the resulting solution was transferred and diluted with 10 mL ultrapure water. The mineralized samples were then diluted with ultrapure water, and each element was analyzed by Inductively Coupled Plasma–Optical Emission Spectroscopy (ICP-OES) (Spectro Amatek Arcos II ICP-OES, Kleve, Germany). The analyses were performed in triplicate.

### 4.5. Analysis of Soluble Proteins, Chlorophyll Content and Total Phenols

The total content of proteins was measured in leaves (1 g) from three individual plants (biological replicates) per treatment, being estimated via the Bradford method and expressed as mg protein g^−1^ FW [55]. Chlorophyll a and b were determined photometrically in leaves according to Ertani et al. [54]. Soluble phenolic acids were extracted by crushing leaves (1 g) in a mortar in the presence of 3 mL pure methanol. The extracts were maintained in an ice bath for 30 min and centrifuged at 5000× *g* for 30 min at 4 °C. The supernatants were stored at −20 °C until analysis. Total phenols were measured according to Arnaldos et al. [56].

### 4.6. Lipid Peroxidation and Antioxidant Enzyme Activities

Lipid peroxidation was carried out by detecting the malondialdehyde content (MDA) via the reaction of thiobarbituric acid (TBA) [57]. Frozen leaf and root tissue (100 mg) samples were ground using a tissue lyser (Biosigma), added with 1 mL of 10% (*w*/*v*) trichloroacetic acid (TCA), and then centrifuged at 13,000 rpm for 10 min at 4 °C. Sample extracts (0.5 mL) were added with 1.5 mL of 0.5% (*w*/*v*) thiobarbituric acid (TBA) in 10% (*w*/*v*) TCA. The samples were incubated in a water bath at 95 °C for 25 min. After that, the reaction was stopped in ice, and samples were centrifuged at 13,000 rpm and 4 °C for 5 min. The absorbance of the samples was measured at wavelengths of 532 nm, 600 nm, and 440 nm, where 532 nm corresponds to the maximum absorbance of TBA-MDA complexes, 600 nm serves as a correction factor for non-specific turbidity, and 440 nm corrects for interference generated by sucrose. The molar extinction coefficient used in the calculations was 157 mM^−1^ cm^−1^.

Guaiacol peroxidase (GPX) and ascorbate peroxidase (APX) activities were measured in root tissues. Enzymes were extracted by homogenizing 200 mg of frozen tissue in 1.5 mL 0.1 M phosphate buffer (pH 7). The homogenate was centrifuged at 15,000× *g* for 5 min at 4 °C, and then the supernatant was collected. GPX activity was determined following guaiacol oxidation with H_2_O_2_ [58]. Shortly, 3 mL guaiacol was added with 20 μL extracts, in the presence of 10 μL H_2_O_2_ (30 vol.). The guaiacol oxidation (ε = 26.6 mM^−1^ cm^−1^) reaction was followed spectrophotometrically as the increase of absorption at 470 nm.

For APX, the activity was determined following the decrease in ascorbate (extinction coefficient 2.8 mM cm^−1^) and measuring the change in absorbance at λ = 290 nm over a 3 min interval. The reaction mixture contained 50 mM phosphate buffer (pH 7.0), 1 mM ethylenediaminetetraacetic acid disodium salt (EDTA)-Na, 0.5 mM ascorbic acid, 0.1 mM H_2_O_2_, and 50 μL of enzyme extract [59]. Three biological replicates from roots of plants of each experimental condition were analyzed.

### 4.7. Statistical Analysis

For all determinations, the analysis of variance (ANOVA) was performed using the R software (4.3.0) and was followed by pair-wise post hoc analyses (Student–Newman–Keuls test) to determine which means differed significantly at *p* < 0.05 (±SD). The number of biological replicates varied depending on the analysis performed and is indicated in the figures and tables’ legends [60].

## 5. Conclusions

The current scarcity of farm strategies designed to mitigate abiotic stresses provides an opportunity for the adoption of biostimulants, or microorganisms, with the potential to enhance plant growth and/or health.

The findings of this study demonstrate that the use of the microalgae-based product was effective in promoting the growth and enhancing its nutritional quality, particularly by increasing the protein content and antioxidant levels. The abundant presence of certain compounds in the MR formulation was likely a key factor contributing to its positive effects on lettuce growth, both under normal conditions and in the presence of salinity.

When plants were subjected to NaCl, the microalgae-based product reduced stress indirectly by restricting Na^+^ accumulation in plant tissues. Perhaps, the content in certain lipid fractions, known for their role in mitigating oxidative stress, may have directly contributed to the observed reduction in lipid peroxidation in NaCl-treated plants primed with the microalgae-based product. We can thus support the use of microalgae as a robust tool for improving the productivity of lettuce under both non-stress and salinity stress conditions through their biostimulant effects. The integration of microalgae-based biostimulants into agricultural practices may offer a promising strategy for sustainable crop production, contributing to improved plant health and resilience in challenging growing environments.

## Figures and Tables

**Table 1:** Elemental composition of microalgae-based product (mean value, ±SD, n = 3).

Parameters	Concentration
TOC (g kg^−1^)	30 ± 2.90
TKN (g kg^−1^)	70 ± 1.78
P (mg kg^−1^)	749 ± 37.84
K (mg kg^−1^)	2452 ± 38.8
Ca (mg kg^−1^)	190 ± 8.70
Mg (mg kg^−1^)	231 ± 9.81
Cu (mg kg^−1^)	1.76 ± 0.38
Zn (mg kg^−1^)	2.23 ± 0.18
Fe (mg kg^−1^)	63 ± 3.82
Mn (mg kg^−1^)	2.10 ± 0.11
pH	3.8 ± 0.06
Moisture (%)	8.84 ± 0.07
Ash (%)	6.75 ± 0.20

Abbreviations. TOC: total organic carbon; TKN: total nitrogen.

**Table 2:** Total lipid contents (% dry weight, DW) and lipid class distribution (% total lipids) of microalgae-based product (mean value, ±SD, n = 3).

Parameters	Concentration
Total lipid	11.11 ± 0.10
MAG	2.91 ± 0.02
DAG	38.51 ± 0.18
TAG	0.84 ± 0.08
FFA	42.31 ± 0.17
HYCA	14.67 ± 0.11
PSTOC	0.75 ± 0.01
Total lipid	11.11 ± 0.10

Abbreviations. MAG: monoacylglycerols; DAG: di-acylglycerols; TAG: triacylglycerols; FFA: phytosterols + tocopherols; HYCA: hydrocarbons + carotenes; PSTOC: free fatty acids.

**Table 3:** Amino acid content of microalgae-based product (MR). Values are the average of three replicates (average ± SD).

Amino Acid	% (g/g)
Glutamic acid	0.43
Leucine	0.42
Alanine	0.38
Valine	0.31
Lysine	0.29
Isoleucine	0.26
Threonine	0.26
Serine	0.22
Histidine	0.19
Asparagine	0.17
Glycine	0.14
Phenylalanine	0.12
Tryptophan	0.10
Methionine	0.10
Aspartic acid	0.09
Arginine	0.07
Tyrosine	0.07
Proline	0.07
Cysteine	0.04
Glutamine	0.03

**Table 4:** Total root length, surface area, number of root tips, forks, crossings, and length of fine roots of untreated plants and MR treated plants. Values are the average of three replicates (average ± SD). Different letters in the same column indicate significant differences between treatments (*p* < 0.05).

Parameters	C	MR	% Increase
Projected Area (cm^2^)	47.34 ± 12.22 ^a^	53.49 ± 8.28 ^a^	113
Surface Area (cm^2^)	148.73 ± 24.80 ^b^	168.05 ± 23.1 ^a^	114
Average Diameter (mm)	0.74 ± 0.046 ^b^	0.57 ± 0.05 ^a^	77
Root Volume (cm^3^)	2.74 ± 0.14 ^b^	2.38 ± 0.09 ^a^	87
Tips	942 ± 23 ^b^	1186 ± 38 ^a^	126
Forks	6254 ± 56 ^b^	7990 ± 37 ^a^	128
Crossings	942 ± 57 ^b^	1568 ± 67 ^a^	166

**Table 5:** Concentration and K^+^/Na^+^ in leaves and roots of *Lactuca sativa* L. plants grown for 14 days in a complete culture solution with 0 (control), 50, and 100 mM NaCl and MR. All data represent the means of five measurements per treatment with three plants in each (±SD). Different letters along each column indicate significant differences between treatments.

	K^+^	Na^+^	K^+^/Na^+^
Leaf	mg kg^−1^ D.W.		
C	7.74 ± 0.82 ^c^	1.96 ± 0.21 ^c^	3.95 ± 0.20 ^a^
MR	7.61 ± 0.38 ^c^	1.87 ± 0.06 ^c^	4.07 ± 0.31 ^a^
NaCl 50	8.99 ± 0.11 ^b^	3.33 ± 0.22 ^a^	2.70 ± 0.11 ^d^
NaCl 100	9.42 ± 0.15 ^b^	3.17 ± 0.20 ^a^	2.97 ± 0.10 ^d^
MR + NaCl 50	9.17 ± 0.12 ^b^	2.54 ± 0.12 ^b^	3.54 ± 0.23 ^c^
MR + NaCl 100	10.24 ± 0.55 ^a^	2.37 ± 0.13 ^b^	4.32 ± 0.27 ^a^
Root			
C	10.03 ± 0.97 ^a^	3.02 ± 0.41 ^c^	3.33 ± 0.32 ^a^
MR	9.60 ± 0.53 ^a^	3.03 ± 0.05 ^c^	3.17 ± 0.13 ^a^
NaCl 50	9.06 ± 0.16 ^b^	4.10 ± 0.25 ^a^	2.21 ± 0.09 ^c^
NaCl 100	8.27 ± 0.12 ^c^	4.28 ± 0.19 ^a^	1.93 ± 0.23 ^c^
MR + NaCl 50	10.29 ± 0.18 ^a^	3.56 ± 0.09 ^b^	2.89 ± 0.13 ^b^
MR + NaCl 100	9.94 ± 0.85 ^a^	2.94 ± 0.10 ^c^	3.38 ± 0.22 ^a^

## Data Availability

Data are contained within the article and Supplementary Materials.

## Supplementary Materials

The following supporting information can be downloaded at https://www.mdpi.com/article/10.3390/plants13233311/s1.
