# Supplementary material for: Mitigation of Salt Stress in Lactuca sativa L. var. Gentile Rossa Using Microalgae as Priming Agents
Source: Plants (Basel). 2024 Nov 26;13(23):3311. doi: 10.3390/plants13233311 (PMC11644400; doi:10.3390/plants13233311)
Supplement: Supplementary file 1 [file plants-13-03311-s001.zip › plants-3298528-supplementary.pdf]

Supplementary Information

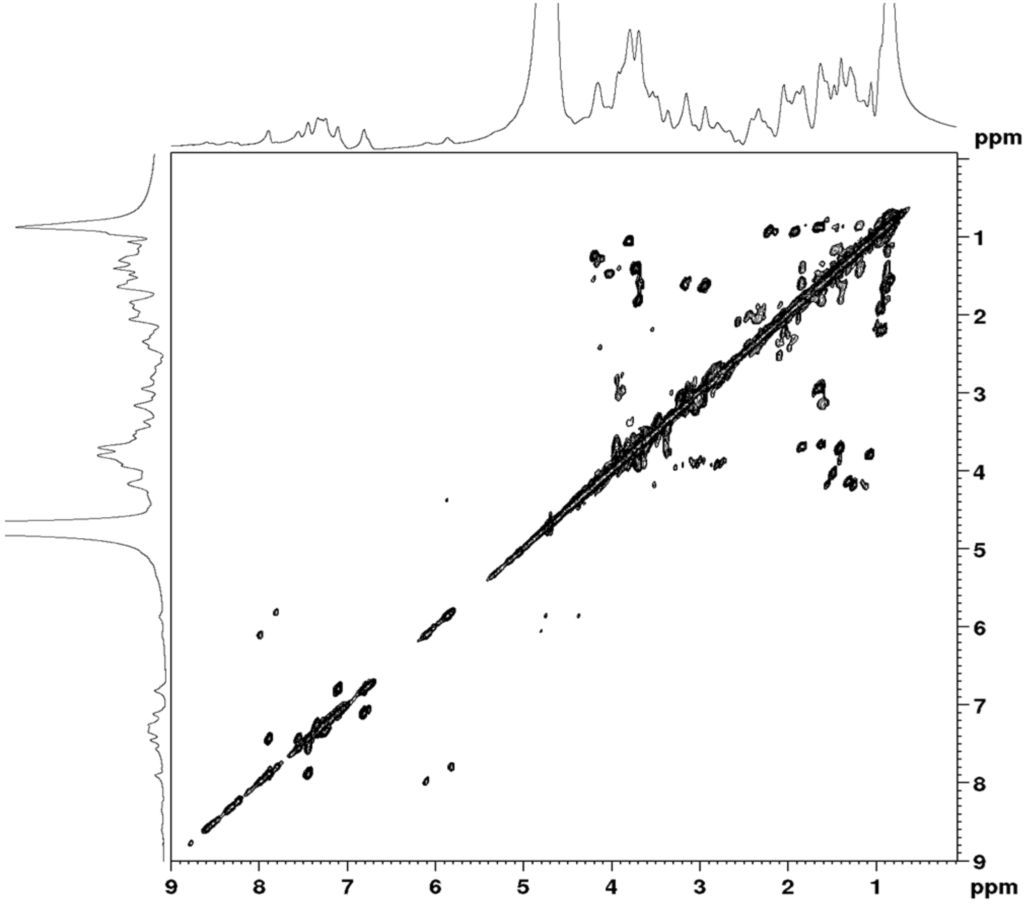

Figure S1NMR. $^{1}H,^{1}H$ COSY spectrum of microalga sample in D_2O (298K @ 600 MHz).

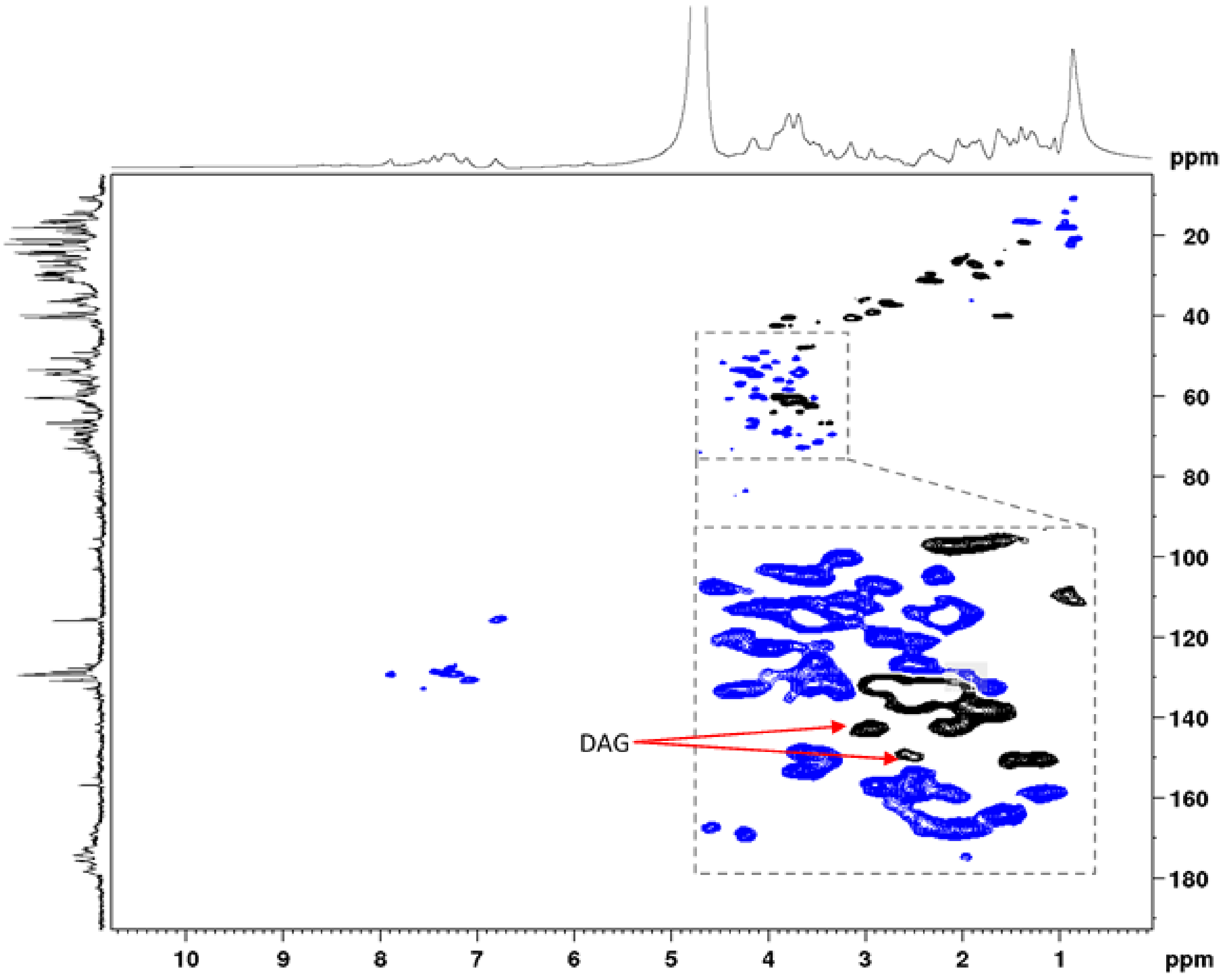

Figure S2NMR. Phase sensitive $^{1}H,^{13}C$ HSQC spectrum of microalga in D_2O at 298 K (@600MHz). Crosspeaks are blue for CH_2 and black for CH/CH_3 groups, respectively. Inset enlarges carbohydrate region that is overlapped with glycerols; red arrows point to crosspeaks tentatively attributed to DAG.

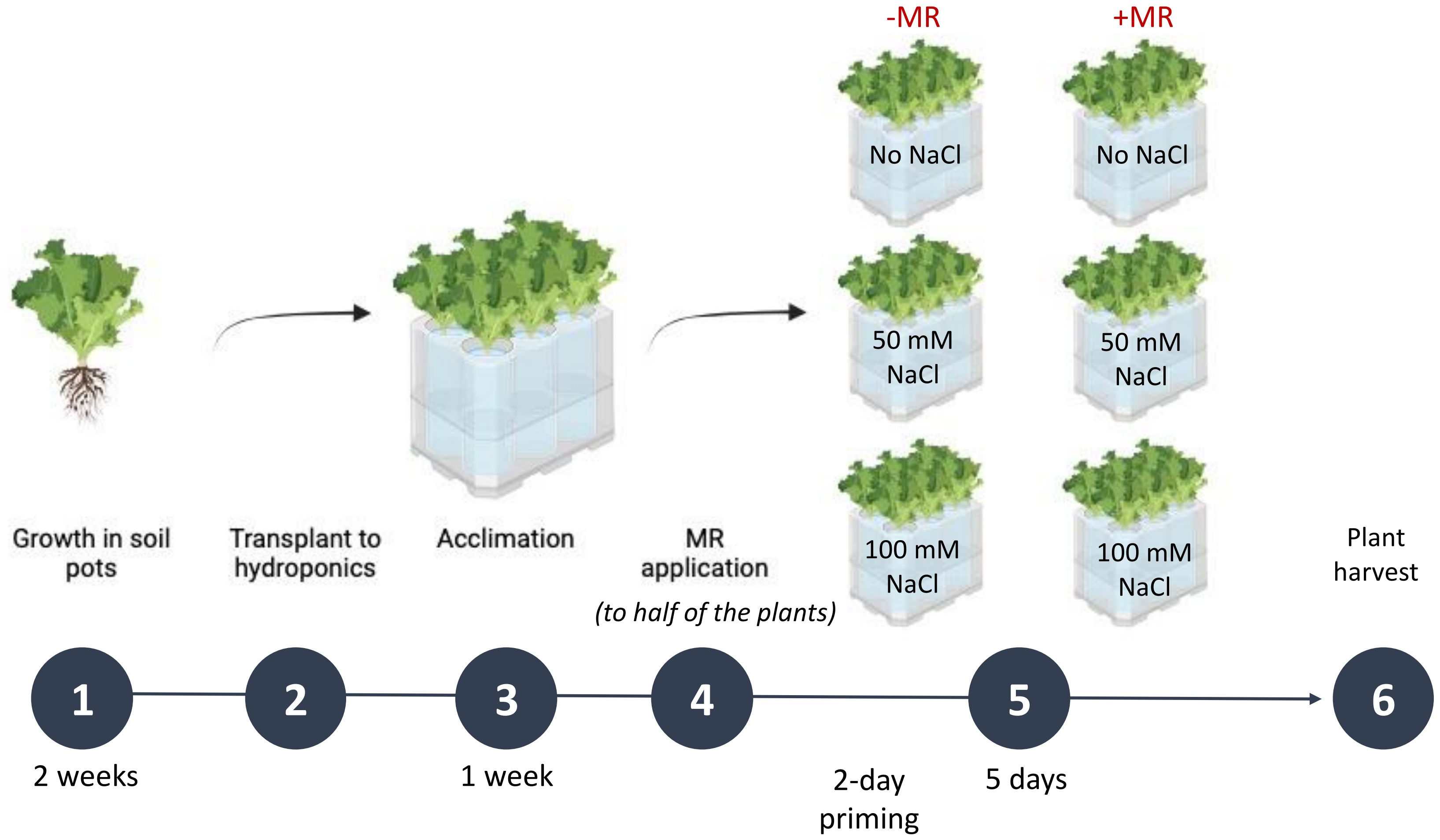

Figure S3. Experimental design.
